# Public health communication and information quality of knee osteoarthritis–related short videos on TikTok, Kwai, and Bilibili: a cross-sectional study

**DOI:** 10.3389/fdgth.2026.1868584

**Published:** 2026-09-28

**Authors:** Yundong Xu, Qianqian Yang, Jian Zhang, Jin Xu, Niqin Xiao, Heguo Yan, Sanjin Zeng, Shengyi Zhao, Rong Wang, Zhaohu Xie, Zhaofu Li

**Affiliations:** Yunnan University of Chinese Medicine, Kunming, Yunnan, China

**Keywords:** China, health communication, information dissemination, knee osteoarthritis, social media, video recording

## Abstract

**Background:**

Knee osteoarthritis (KOA) is a prevalent musculoskeletal disorder and a major cause of pain and disability. As short-video platforms increasingly shape how the public accesses health information, concerns have grown regarding the quality, reliability, and completeness of KOA-related content. This study evaluated KOA-related videos on three major Chinese short-video platforms, TikTok, Kwai, and Bilibili, to compare information quality, user engagement, and implications for digital public health communication.

**Methods:**

In this cross-sectional study, standardized Chinese keywords were used to retrieve KOA-related videos from TikTok, Kwai, and Bilibili on December 24, 2025. After deduplication and screening, 300 videos were included, 100 from each platform. Video characteristics and engagement indicators were recorded. Information quality was assessed using the DISCERN reliability section, Global Quality Score (GQS), content completeness score (CS, range 0–10), and engagement score (ES, range 0–4). Uploaders were categorized by source type. Statistical analyses included the Kruskal–Wallis H test, chi-square test, and Spearman correlation analysis.

**Results:**

Substantial cross-platform differences were identified. Bilibili videos had the longest duration (median 402 s) and the highest information quality, with superior DISCERN scores (median 32), GQS (mean 4.3), and CS (mean 7), and had the highest proportion of certified medical professionals among uploaders (38.0%). TikTok videos were the shortest (median 86 s) but showed the highest user engagement, including likes, comments, shares, and the highest ES (median 3.6). TikTok and Kwai videos were generally less complete, focusing mainly on symptoms and treatment while providing limited information on etiology and prognosis. Correlation analysis showed that higher information quality and completeness were negatively associated with user interaction metrics, whereas ES was positively associated with engagement.

**Conclusions:**

The three major short-video platforms in China represent distinct digital health information ecosystems for KOA, revealing a consistent trade-off between scientific rigor and audience engagement. Platform recommendation mechanisms may may preferentially surface content that is more attractive and interactive rather than more accurate and comprehensive. These findings highlight an important public health challenge in the governance of online health information and suggest that improving chronic disease communication on social media will require both stronger platform-level quality prioritization and more engaging evidence-based content produced by health professionals.

## 1 Introduction

Knee osteoarthritis (KOA) is a chronic degenerative joint disease that is a leading cause of pain and lower limb disability among middle-aged and elderly populations globally (1). The rapid acceleration of population aging has intensified the disease burden associated with KOA (2, 3). This trend is particularly severe in China, where the prevalence of KOA is reported at 8.1% (4), affecting more than 100 million individuals—a figure projected to rise alongside increasing life expectancy (5, 6). The primary clinical threats posed by KOA include progressive articular cartilage wear and functional loss (7), resulting in long-term pain and significant economic burden (8). Although no specific cure currently exists, international clinical guidelines unanimously emphasize that patient education—encompassing weight management (9), scientific exercise therapy, and lifestyle interventions—is critical for delaying disease progression, relieving pain, and improving joint function (10). However, pervasive misconceptions regarding disease management remain common, such as the dichotomy between absolute sedentary behavior and excessive exercise (11). These cognitive biases frequently lead to clinical deterioration and unnecessary medical resource consumption, underscoring the pivotal role of high-quality health education in the management of chronic KOA (12).

The mechanisms by which the public acquires health information have undergone a fundamental shift in the era of ubiquitous mobile internet access. Online sources are frequently consulted before or alongside professional healthcare, and it has been reported that approximately 80% of Internet users obtain medical information online (13). Social media, particularly short-video platforms, has emerged as a primary channel for medical knowledge dissemination due to its accessibility, visual intuitiveness, and fragmented content delivery. In China, three major platforms—TikTok, Kwai, and Bilibili—have hundreds of millions of active users. Notably, a significant demographic overlap exists between the aging population, who are increasingly becoming loyal users of short-video platforms, and the high-incidence group for KOA. These platforms can accurately push disease-related content to potential patients through algorithmic recommendations, thereby facilitating information acquisition (14). However, the quality of content on these platforms is inconsistent; a substantial volume of misinformation, ranging from unverified “miracle medicines” to pseudoscientific exercise guidance, lacks a scientific basis (15). For patients with limited health literacy, uncritical adherence to such content may not only fail to improve their condition but also worsen joint damage and increase health risks (16). This concern is particularly relevant for short videos because medical messages are delivered not only through speech but also through subtitles, captions, and other written elements. Online information may also shape how patients understand disease causes, pathophysiology, symptoms, and treatment options, thereby influencing treatment expectations, self-management behavior, and decision-making (17, 18).

Recently, the academic community has increasingly focused on the role of social media in the management of chronic diseases. Bibliometric and Altmetric approaches have further suggested that social media interaction can complement traditional citation indicators by reflecting academic visibility and the dissemination of medical literature. While studies have evaluated the quality of arthritis-related videos on international platforms such as YouTube or TikTok (15), systematic research regarding China's digital environment remains insufficient. The importance of online platforms has also been highlighted during public-health crises, when users and healthcare professionals often seek rehabilitation information or treatment-protocol guidance online (19). China's three mainstream short-video platforms exhibit distinct operational characteristics: TikTok emphasizes algorithmic recommendations, Kwai focuses on community interaction, and Bilibili is characterized by medium- to long-form content (20). These structural differences may may be associated with differences in the quality and completeness of health information provided on each platform (21). Previous Chinese short-video studies have examined health information quality in other diseases, including brain tumors and lung cancer (21, 22), but evidence specific to KOA across TikTok, Kwai, and Bilibili remains limited.

These three platforms were selected because they represent major but structurally different short-video ecosystems in China, whereas platforms such as Xiaohongshu, YouTube, Instagram, and Facebook differ in access environment, content format, user base, or platform governance and were beyond the scope of the present China-focused comparison. Accordingly, a combined assessment of KOA-related information reliability, content completeness, uploader source, audience-oriented engagement, and user interaction across these platforms remains warranted.

This study addresses a digital public health problem by examining whether high-exposure short-video content provides sufficiently reliable and complete information to support public understanding and self-management of a prevalent chronic disease. Using a cross-sectional design, we systematically evaluated the quality, credibility, completeness, and engagement characteristics of KOA-related videos on TikTok, Kwai, and Bilibili (23). Specifically, we compared platform-level differences in information quality and uploader characteristics and explored the relationship between scientific quality and user engagement (24). By incorporating the dimensions of readability, health literacy, platform-specific communication patterns, and user decision-making, this study aims to provide evidence for improving digital health communication, strengthening public health messaging, and informing governance strategies for online health information in China (25).

## 2 Materials and methods

### 2.1 Research design and search strategy

This study used a cross-sectional design to systematically evaluate the content quality of videos related to knee osteoarthritis on Bilibili, Kwai, and TikTok. All data were collected uniformly on December 24, 2025 to reduce the time deviation caused by platform content updates and fluctuations in the sorting algorithm. Information such as terminal type, operating system, application version, and network environment used is synchronized during the acquisition process to ensure that the same terminal and network settings are used throughout the acquisition process.

The study uses two Chinese keywords, “knee osteoarthritis” and “knee arthritis”, and if the platform automatically associates the keywords with the topic page, the search results presented by the platform by default will prevail. In the medical context, the two usually refer to the same disease. However, given the differences in habits across different regions, ages, and internet terminology, the use of a single term may result in incomplete sample coverage. Therefore, a two-keyword strategy was adopted to reduce the resulting sampling bias.

The search results of each platform were collected according to the default platform ranking, without secondary sorting or manual adjustment, to approximate the content exposure most likely to be encountered by ordinary users in real-world searching. For each platform, the top 50 videos under the default ranking were first collected using “膝骨关节炎” as the keyword, and additional nonduplicate videos were then supplemented using “膝关节炎” until 100 eligible videos were obtained for each platform. Because default ranking may be influenced by proprietary recommendation algorithms, all searches were conducted within the same time window using newly created accounts, identical device and network conditions, and no prior interactive behavior, in order to reduce personalization bias and improve cross-platform comparability (26, 27). Geographical metadata were not consistently available across the three platforms and were therefore not included as a comparative variable in the present study.

### 2.2 Deduplication, eligibility criteria, and bias reduction

Video deduplication was based on “the same video on the same platform.” The decision was made according to the unique video link or ID displayed on the platform. If this information was not visible, duplicate status was determined using the title (or first-sentence copy), publisher account, video duration, and cover-screen similarity. Videos were not deduplicated across different platforms.

All included videos were in Chinese or in English with accurate Chinese subtitles, so that the general Chinese audience could understand and evaluate the information comparably. Videos were excluded if they were duplicate videos within the same platform keyword search, English-only videos without Chinese subtitles, videos without audio or without sufficient text instructions to judge their medical information, content not directly related to knee OA, or nonstandard entries such as live streams, collection pages, unplayable videos, or removed content. Videos meeting any of these exclusion criteria were not included in the final analysis (Figure 1). After deduplication and screening, 300 videos were ultimately included for subsequent statistical analysis and quality evaluation.

**Figure 1:**
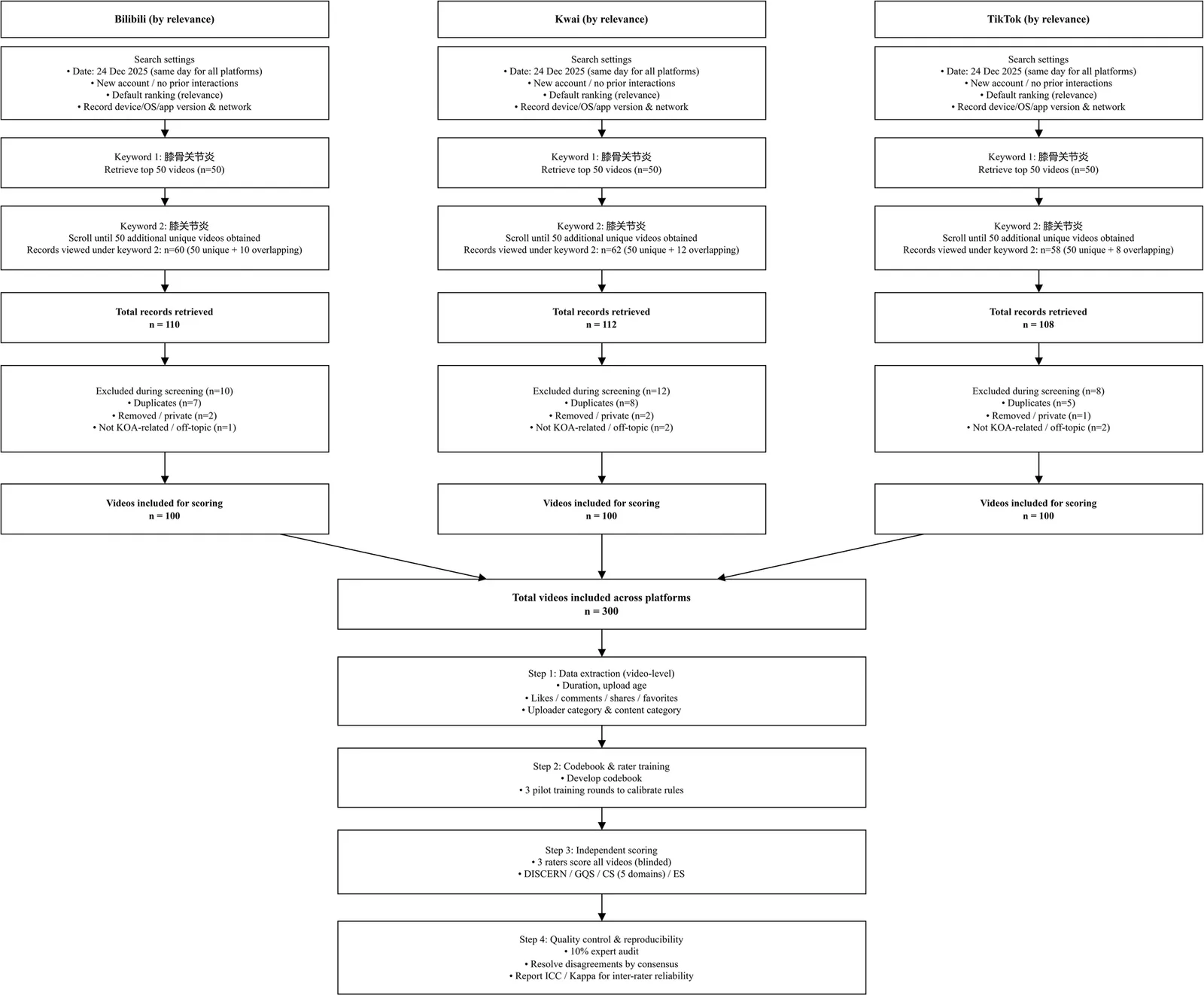
Flowchart of the retrieval, screening, and inclusion process of KOA-related videos on TikTok, Kwai, and Bilibili.

To reduce the influence of platform personalization and historical behavioral data, newly created accounts were used for data collection on all three platforms. Before the formal search, no interactive behaviors, such as following, liking, commenting, favoriting, or forwarding, were performed. Only the predefined research keywords were used in the search process, and no additional health or non-health topic content was searched, in order to improve the reproducibility and external validity of the results.

### 2.3 Video feature extraction and variable definition

We extracted and recorded the visible characteristics and engagement metrics of each included video, including video duration (seconds) and upload date when available. These variables were used to describe the sampled content and to provide temporal context for the retrieved videos. The number of likes, comments, shares, and favorites is indicated in the variable table to ensure cross-platform comparability. The above variables describe the overall characteristics of the sample and support the comparative analysis of the information quality and engagement performance of different platforms and uploader categories.

### 2.4 Video content, presentation type, and author classification

To more comprehensively portray the content ecology of KOA short videos, we additionally classify the types of video presentations as follows: popular science, professional courses, personal experience sharing, and others (such as advertising, entertainment, etc., which lack effective medical information). The classification is based on the comprehensive judgment of the video body content and the account positioning information. When boundary situations are encountered, they are handled according to the coding manual's example rules, and the caliber is unified after discussion within the group.

Based on the framework of previous research, we initially divided uploaders into seven categories: certified medical professionals, patients or dependents, general users, science communicators, news organizations, nonprofits, and for-profit organizations. The judgment of the uploader is based on comprehensive judgments of visible information, such as platform authentication logos, account profiles, institutional qualification displays, and content self-declaration. Owing to the small sample size of some categories (<5), to improve the stability of the statistical analysis and follow conceptual similarity, we merged the categories and merged “patients or family members” into “ordinary users”. Merge “news organization”, “nonprofit organization”, and “for-profit organization” into “organization”. The final statistics include four types of uploaders: certified medical professionals, ordinary users, science communicators, and organizations. Details of the uploader classification rules and judgment criteria are provided in the Supplementary Materials (Supplementary Table S1).

### 2.5 Coding and quality control

Before the formal evaluation began, the research team developed a unified coding manual through multiple discussions, clarifying platform feature variables, uploader category judgment rules, video type classification, and each scoring tool's operational definitions. Moreover, the researchers conducted three rounds of precoding training to improve the consistency between raters. After scoring the same batch of samples independently, they compared the differences and discussed them intensively to gradually clarify the vague and confusing items and ensure that the scoring rules were uniformly enforced. Three researchers (XYD, YQQ, and ZJ) independently coded and evaluated the quality of all included videos without knowing each other's scoring results. Two senior experts (XNQ and YHG) randomly selected 10% of the samples for review to further evaluate the accuracy and consistency of the coding. If disagreements arose, consensus was reached through discussion to determine the final value, and key divergence types were recorded in the coding manual to reduce subsequent bias. In addition, the interrater agreement index for the core continuous score (DISCERN, GQS, CS, ES total score) and the agreement index (Kappa coefficient) for the categorical variables (uploader type, video presentation type, etc.) were calculated. These procedures were used to improve the internal consistency and reproducibility of the adapted coding framework in the context of Chinese short-video assessment.

### 2.6 Assessment of video quality

The reliability section of the DISCERN tool was used to evaluate the reliability of video information. Because DISCERN was originally developed for English-language written consumer health information, its use in this study required operational adaptation to the context of Chinese short-video content. Specifically, each DISCERN item was translated into observable short-video indicators, and detailed scoring anchors were developed for use in Chinese-language KOA-related videos. These operational definitions and scoring criteria are provided in Supplementary Table S2. The adapted scoring framework was refined through repeated team discussion and three rounds of precoding before formal evaluation. This section contains 8 items, each scored on a scale of 1–5, with higher scores indicating greater reliability. To ensure that the items are consistent in direction (28), reverse scoring is clearly and uniformly implemented in the coding manual if there are items on the scale that need to be understood in reverse (such as items related to bias and uncertainty) (29). The average item score (1–5) and the total score (8–40) were both reported for statistical presentation, and the same scoring standard was maintained throughout the study (30).

The overall quality and educational value of the videos were evaluated using the GQS. The GQS is a 5-point scale (1–5 points), with higher scores indicating better overall video quality (31).

Information integrity is assessed using a specially developed CS checklist. The list contains five core areas: causes/risk factors, symptoms, prevention, treatment, and prognosis (32). Each domain is scored on a scale of 0–2 (0 = not mentioned, 1 = briefly mentioned, 2 = detailed), with a total score ranging from 0 to 10 points. For ease of interpretation, the total score can be divided into the following levels: 0–1 (almost no valid information), 2–3 points (more missing information), 4–5 points (missing some information), 6–7 points (less missing information), 8–9 points (covering most of the key information), and 10 (complete coverage and sufficient explanation) (33).

User engagement is measured using the ES (34). ES contains two dimensions, comprehensibility (0–2 points) and entertainment/attractiveness (0–2 points), with a total score ranging from 0 to 4 points. The comprehensibility dimension evaluates the clarity and ease of understanding of the content expression, and the specific scores are as follows: 2 points (clear explanation and easy-to-understand scientific content), 1 point (limited understanding due to too many technical terms), and 0 points (obscure content or almost copying literature). The entertainment/attractiveness dimension evaluates the presentation's attractive effect on the audience and is divided into 2 points (both popular science and attractiveness, good viewing experience), 1 point (limited entertainment), and 0 points (boring and unattractive presentation). The total ES score was used to analyze the correlation with video engagement data and other quality metrics. The Supplementary Materials (Supplementary Table S3) provide specific explanations and examples of grading scales (35).

### 2.7 Statistical analysis

All statistical analyses were performed using SPSS 26.0, and normality was assessed using the Shapiro–Wilk test. The normally distributed variables are expressed as the mean ± standard deviation, and those that were not normally distributed are expressed as the median (interquartile range, IQR). Categorical variables are expressed as frequencies (percentages). For comparisons between groups, the KruskalWallis H test was used to analyze the differences in continuous variables between different platforms or uploader types. If there was a significant difference, a twobytwo comparison was performed, and the *P* value was corrected using the Bonferroni method. Differences in the distribution of categorical variables were evaluated using the chi-square test, *post hoc* comparisons were performed, and column proportions were multicorrected if necessary. Spearman rank correlation analysis was used to explore the correlation between video characteristics (e.g., duration, number of upload days, and number of likes) and scoring indicators (e.g., DISCERN, GQS, CS, and ES). All statistical tests were two-tailed, and a *P* value of 0.05 was considered statistically significant.

## 3 Results

### 3.1 Overview of the search strategy and sample inclusion

Following a standardized dual-keyword search strategy (“膝骨关节炎” and “膝关节炎”), data retrieval and screening were synchronized across the three platforms within the same time window (Figure 1). The top 50 videos ranked by the platform's default relevance algorithm were initially retrieved for each keyword. After merging the results, removing duplicates, and applying predefined exclusion criteria—including removed, private, or non–KOA-related content—a final homogeneous sample of 300 videos was established, comprising 100 videos from each platform (Bilibili, Kwai, and TikTok).

This balanced sample distribution across platforms provided a robust foundation for subsequent cross-platform comparisons of content characteristics, information quality, and user engagement.

### 3.2 Video morphological characteristics: duration heterogeneity and interaction indicators

Descriptive statistical analysis revealed that the video duration and user interaction behavior of different platforms presented significant dichotomous differentiation characteristics (Table 1). In terms of time, Bilibili videos were significantly longer than those of other platforms, with a median duration of 402 s (IQR 349–460), far exceeding those of Kwai (110 s, IQR 90–130) and TikTok (86 s, IQR 73–99), and the difference was statistically significant (*H* = 212.74, *P* < 0.001).

**Table 1:** Detailed characteristics of KOA-related videos across platforms.

Parameters	Bilibili (*N* = 100)	Kwai (*N* = 100)	TikTok (*N* = 100)	H/*χ*^2^ (*P* value)
Likes, median (IQR)	932 (724–1,219)	2,003 (1,538–2,730)	5,173 (3,396–6,838)	216.88 (<0.001)
Comments, median (IQR)	80 (58–107)	166 (117–240)	434 (280–624)	196.13 (<0.001)
Shares, median (IQR)	50 (35–67)	112 (76–148)	281 (189–410)	202.62 (<0.001)
Duration (seconds), median (IQR)	402 (349–460)	110 (90–130)	86 (73–99)	212.74 (<0.001)
Video source				χ²=18.13 (*P* = 0.006)
Medical professionals, *n* (%)	38 (38.0)	25 (25.0)	23 (23.0)	
General users, *n* (%)	16 (16.0)	25 (25.0)	37 (37.0)	
Science communicators, *n* (%)	28 (28.0)	26 (26.0)	29 (29.0)	
Organizations, *n* (%)	18 (18.0)	24 (24.0)	11 (11.0)	
Information quality				
DISCERN score (8–40), median (IQR)	32 (29–35)	19 (17–22)	23 (19–25)	186.84 (<0.001)
GQS score (1–5), median (IQR)	4.3 (4.0–4.6)	2.9 (2.5–3.3)	3.1 (2.9–3.5)	185.05 (<0.001)
Completeness score (0–10), median (IQR)	7 (7–8)	4 (3–5)	4 (3–5)	168.64 (<0.001)
Engagement score (0–4), median (IQR)	2.4 (2.2–2.7)	2.6 (2.3–2.9)	3.6 (3.4–3.8)	192.33 (<0.001)

Continuous variables are presented as median (IQR). H denotes the Kruskal–Wallis test statistic; χ² denotes the chi-square test statistic. *P* values are two-sided; *P* < 0.05 was considered statistically significant. KOA, knee osteoarthritis; GQS, Global Quality Score; CS, Completeness Score; ES, Engagement Score; IQR, interquartile range.

In terms of interaction, TikTok demonstrated significantly higher engagement metrics than the other platforms in key interaction indicators such as likes (median 5173), comments (434), and shares (281), and significantly better than Kwai (2003, 166, 112, respectively) and Bilibili (932, 80, 50, respectively) (the H value is between 196.13 and 216.88, *P* < 0.001). The above data show that Bilibili videos are mainly long-form videos with relatively limited interaction, whereas TikTok videos are highly popular in the form of short videos.

### 3.3 Differences in uploader distribution on different platforms

The distribution of uploader types differed significantly among different platforms (*χ*^2^ = 18.13, *P* = 0.006). BiliBili has the highest proportion of certified medical professionals (38.0%), demonstrating its professional content aggregation characteristics. In contrast, the TikTok platform has the highest proportion of ordinary users (37.0%), reflecting a more popular content ecosystem. The Kwai platform accounted for a high proportion of organizational accounts (24.0%). In addition, the proportion of science communicators on each platform is relatively close, ranging from 26.0% to 29.0%, indicating that science popularizers have become a stable force in the field of health science popularization (Table 2).

**Table 2:** Platform comparison of DISCERN, GQS, CS, and ES scores for KOA-related videos.

Parameters	Bilibili (*N* = 100)	Kwai (*N* = 100)	TikTok (*N* = 100)	H (*P* value)
DISCERN score (8–40), median (IQR)	32 (29–35)	19 (17–22)	23 (19–25)	186.84 (<0.001)
GQS score (1–5), median (IQR)	4.3 (4.0–4.6)	2.9 (2.5–3.3)	3.1 (2.9–3.5)	185.05 (<0.001)
Completeness score (0–10), median (IQR)	7 (7–8)	4 (3–5)	4 (3–5)	168.64 (<0.001)
Engagement score (0–4), median (IQR)	2.4 (2.2–2.7)	2.6 (2.3–2.9)	3.6 (3.4–3.8)	192.33 (<0.001)

Scores are presented as median (IQR). H denotes the Kruskal–Wallis test statistic. *P* values are two-sided; *P* < 0.05 was considered statistically significant. KOA, knee osteoarthritis; GQS, Global Quality Score; CS, Completeness Score; ES, Engagement Score; IQR, interquartile range.

### 3.4 Comparison between information quality and user engagement platforms

There was a clear differentiation among platforms in terms of information quality and user engagement. BiliBili is significantly ahead in terms of indicators reflecting information rigor, and its information reliability (median DISCERN score of 32), overall quality (GQS mean of 4.3), and content integrity (CS mean of 7) are higher than those of Kwai and TikTok (*P* < 0.001). Among the three platforms, Kwai's DISCERN (median 19) and GQS (average 2.9) scored the lowest.

In terms of the ES, which measures user attractiveness, TikTok scored the highest (median 3.6, IQR 3.4–3.8), followed by Kwai (2.6), and BiliBili scored the lowest (2.4), with a statistically significant difference (*P* < 0.001). This misalignment between quality and appeal indicates that high-quality content often struggles to gain a high level of user engagement. (Figure 2, Table 2).

**Figure 2:**
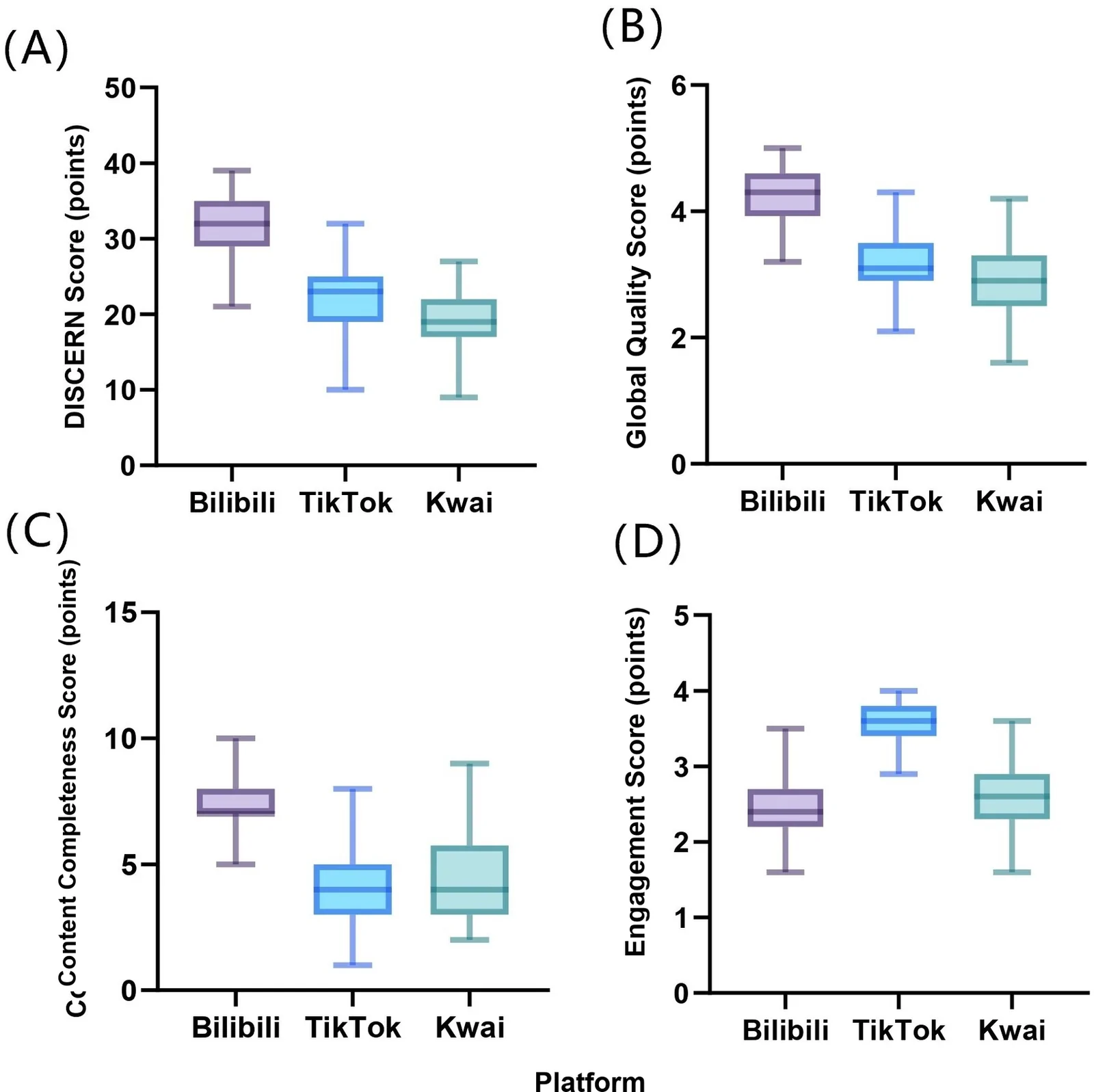
Comparison of information quality (DISCERN, GQS, CS) and engagement (ES) scores of videos across TikTok, Kwai, and Bilibili.

### 3.5 Integrity analysis of the core topic coverage

Further domain coverage analysis revealed structural differences in content depth between platforms. Bilibili scored the highest in overall completeness, achieved relatively balanced “detailed coverage” (score of 2) for each subject, and showed systematic knowledge output ability in terms of treatment (77.0%), symptoms (75.0%), prognosis (48.0%), and etiology (44.0%). In contrast, TikTok and Kwai's content coverage is obviously insufficient. TikTok focuses on symptom description (55.0%), but the coverage rates of etiology (2.0%) and prognosis (10.0%) are low. Although Kwai has a certain focus in the therapeutic field (56.0%), the detailed coverage rate in the other four dimensions is less than 25.0%. (Table 3).

**Table 3:** Domain-level completeness (detailed coverage) for KOA-related videos.

Parametersn (%)	Bilibili (*N* = 100)	Kwai (*N* = 100)	TikTok (*N* = 100)	H/χ^2^ (*P* value)
Etiology	44 (44.0%)	6 (6.0%)	2 (2.0%)	112.50 (<0.001)
Symptoms	75 (75.0%)	18 (18.0%)	55 (55.0%)	81.98 (<0.001)
Prevention	41 (41.0%)	21 (21.0%)	25 (25.0%)	18.47 (0.001)
Treatment	77 (77.0%)	56 (56.0%)	20 (20.0%)	74.06 (<0.001)
Prognosis	48 (48.0%)	17 (17.0%)	10 (10.0%)	147.41 (<0.001)
Total CS (0–10), median (IQR)	7 (7–8)	4 (3–5)	4 (3–5)	168.64 (<0.001)

Domain cells show the proportion of videos with detailed coverage (domain score = 2). Each domain is scored 0–2 (0 = not mentioned, 1 = briefly mentioned, 2 = discussed in detail). Domain comparisons use the chi-square test; Total CS uses the Kruskal–Wallis test (H). KOA, knee osteoarthritis; CS, Completeness Score; IQR, interquartile range.

### 3.6 Correlation between the characteristics of video dissemination and information quality

Spearman's correlation analysis revealed a negative relationship between audience engagement and information quality (Figure 3). The data show a consistent and significant negative correlation between the interaction indicators (likes, comments, and shares) and the quality indicators (DISCERN, GQS, and CS) that represent the content value. Among them, the negative correlation between CS and interaction metrics is the most significant, suggesting that the more comprehensive and detailed the content, the more difficult it is to gain audience attention.

**Figure 3:**
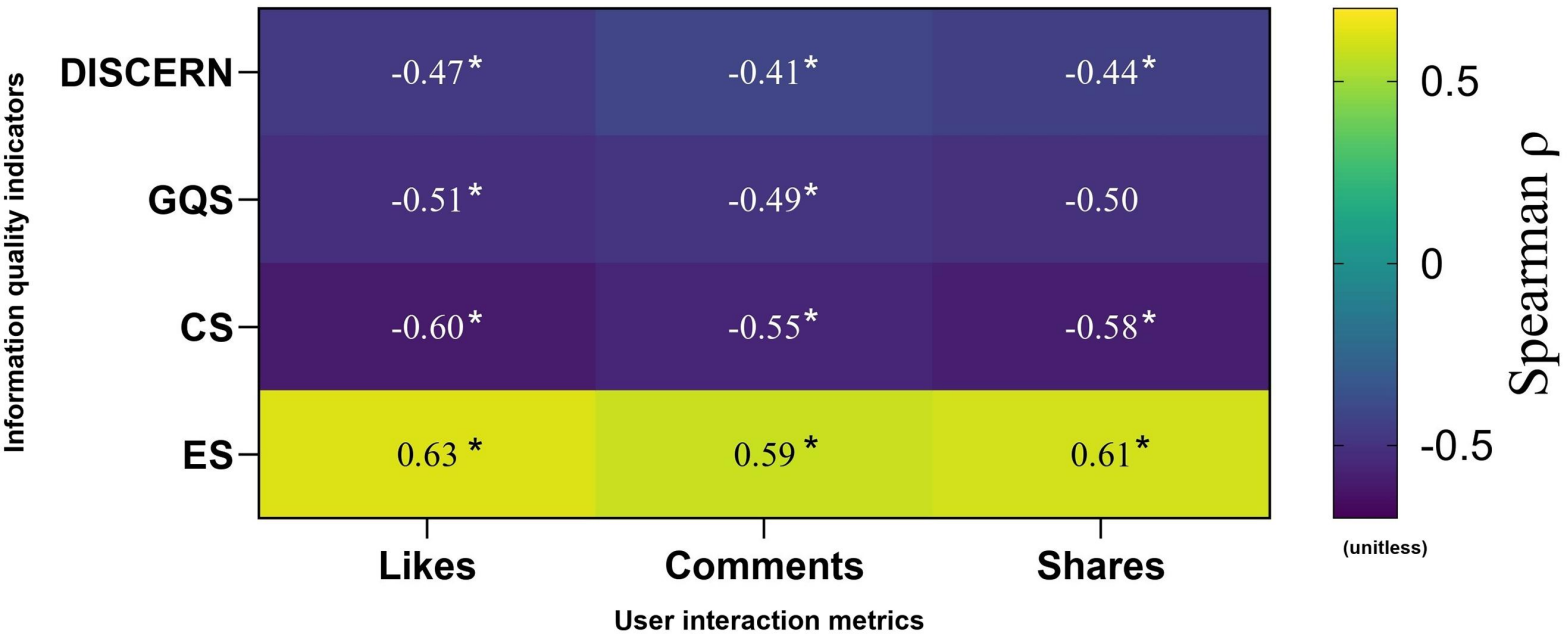
Correlation between video information quality indicators and user interaction metrics on short-video platforms.

In contrast, the ES was strongly positively correlated with each interaction metric. This suggests that the recommendation mechanism of the current platform may be more inclined to promote content that can stimulate immediate interaction and has high attractiveness than popular science works with better information quality or greater comprehensiveness.

## 4 Discussion

### 4.1 Platform heterogeneity and quality stratification in DH communication

This study systematically analyzed the ecological differences in knee osteoarthritis-related content across three short-video platforms using standardized synchronous retrieval and multidimensional evaluation (14). The data indicate that significant disparities exist among the platforms regarding information quality, content comprehensiveness, and user interaction. Bilibili demonstrated superior scientific accuracy, overall educational quality, and content breadth, effectively serving as the primary vehicle for the dissemination of in-depth health knowledge (36). Conversely, while TikTok exhibited a distinct prominence in user engagement and interaction metrics, its content's professional quality was found to be comparatively lower. The observed discrepancy between “high quality–low interaction” and “low quality–high interaction” highlights the inherent tension between scientific rigor and widespread dissemination in digital health communication. Furthermore, these findings may reflect associations between platform characteristics, user demographics, and divergent health information environments (30).

Recent studies on musculoskeletal health information and digital health communication further highlight the importance of evaluating online resources with multidimensional criteria rather than relying only on visibility or popularity (37). These studies have examined large language models' interpretation of evidence-based spine guidelines, prevention-oriented osteoarthritis education, the quality, reliability (38), and popularity of YouTube videos on thoracic outlet syndrome, and the content quality and readability of AI-supported osteoarthritis information texts (39, 40). Together, these findings support the need to assess digital health information in terms of accuracy, reliability, readability, completeness, and audience response. Our study extends this evidence to the Chinese short-video environment for knee osteoarthritis and shows that audience engagement may diverge from scientific completeness and reliability.

### 4.2 Impact of platform media characteristics on content construction

The advantages of Bilibili in terms of information quality and comprehensiveness may be related to its long-form and professionally oriented content ecosystem. Longer videos allow creators to present more structured explanations of disease mechanisms, diagnosis, treatment, and self-management, while the relatively high presence of certified medical professionals may further support content credibility. By contrast, the rapid-consumption communication model of TikTok and Kwai is designed to capture audience attention within shorter timeframes. This format may encourage simplified, emotionally resonant, or immediately practical messages; although such content can improve accessibility, it may also reduce medical context and limit the depth of health education.

### 4.3 Structural imbalances in content coverage and their potential impacts

The observed imbalance in topic coverage suggests that KOA-related short videos may present the disease in a fragmented manner. Previous analyses of TikTok health videos have similarly shown that popular posts often emphasize observable behaviors and easily consumable topics, such as diet, exercise, or symptoms, while providing less guidance on underlying causes, prevention, or long-term management (41). Such patterns may contribute to an incomplete understanding of KOA, with greater attention to immediate symptom relief than to systematic prevention and disease control. In addition, insufficient etiological context may increase the risk of misinterpretation or inappropriate self-management (42) These findings highlight the need for more balanced health communication that integrates symptoms, treatment, risk factors, prevention, prognosis, and appropriate clinical guidance.

### 4.4 The inverse association between audience engagement and information quality

The inverse association between audience engagement and information quality is an important finding of this study. It suggests that engagement indicators such as likes, comments, and shares should not be interpreted as direct markers of medical value. In short-video environments, highly watchable or emotionally appealing content may receive stronger audience responses, whereas scientific depth and informational rigor do not necessarily generate high engagement (43). Evidence-based medical content often requires structured explanation and reference to clinical evidence, which may reduce immediate audience interaction despite its educational value (36). In platform environments that prioritize interaction-driven features, such content may therefore be less likely to achieve high visibility (44). These findings support the use of multidimensional assessment frameworks that combine engagement metrics with quality indicators such as DISCERN, GQS, and content completeness.

### 4.5 Practical implications and optimization directions

This study provides several implications for improving the digital dissemination of health information. At the platform level, recommendation and moderation systems should move beyond purely engagement-driven logics and incorporate stronger quality-oriented criteria for health-related content. In particular, platforms could increase the visibility of evidence-based content produced by certified medical professionals while identifying or flagging unsupported and potentially misleading information, in line with established principles for recognizing credible health information on social media (45). Healthcare professionals and public health institutions should also strengthen their presence on high-traffic short-video platforms and develop segmented, serial, and audience-adapted content that addresses underrepresented domains such as disease etiology, prevention, and prognosis. Such structured and evidence-based communication is essential for countering misinformation and improving public health literacy in social media environments (46). In addition, content development strategies should aim to balance scientific rigor with audience engagement. Creators should clearly communicate the scope, applicability, and limitations of the information they provide to reduce misunderstanding and overgeneralization. They should also adopt formats that connect fragmented messages into more coherent and comprehensive knowledge structures, thereby improving both completeness and scientific integrity. Overall, although adapting to platform-specific dissemination patterns may help increase reach, maintaining medical accuracy, transparency, and professional standards remains essential for delivering credible and effective health communication online (47). The present findings may also be interpreted in a broader multilingual and cross-platform context. Concerns regarding the readability, quality, and reliability of online health information are not limited to Chinese- or English-language environments. For example, a Turkish study on internet-based patient education materials related to low back pain reported important readability limitations, suggesting that the challenge of making digital health information both accessible and clinically appropriate extends across languages and healthcare settings (48). In addition, the platforms examined in the present study differ in important ways from other popular channels. Compared with TikTok and Kwai, YouTube generally supports longer and more searchable video-based education, whereas web-based health channels may provide more structured but less interactive information. By contrast, AI-based tools and conversational systems can deliver on-demand responses and a greater appearance of personalization, but recent studies suggest that their readability, quality, and reliability remain variable (49). These differences indicate that platform form, interaction logic, and information architecture should all be considered when evaluating the public-health value of digital medical information.

### 4.6 Strengths and limitations

This study has several strengths. First, it used a synchronized retrieval strategy across three major short-video platforms within the same time window, which improved comparability across platforms. Second, it combined multiple dimensions of evaluation, including reliability, overall quality, content completeness, uploader source, and user engagement, thereby allowing the study to assess not only what information was presented but also how that information circulated. Third, by focusing on KOA, a highly prevalent chronic condition that requires sustained self-management, the study addresses a clinically meaningful and public-health-relevant topic.

Several limitations should be acknowledged. First, only TikTok, Kwai, and Bilibili were included; therefore, other important channels, such as YouTube, AI-assisted information tools, and general web-based health resources, were not directly evaluated. Second, because the sample was based on platform-visible short videos retrieved within a defined time window and according to default platform ranking, the findings should be interpreted as user-facing visible content rather than a complete representation of all KOA-related videos available on each platform. Although newly created accounts, synchronized searches, and standardized search procedures were used to reduce personalization bias, the influence of proprietary recommendation algorithms cannot be fully excluded. Third, temporal variation in upload patterns was not analyzed in detail, and geographical metadata were not consistently available across platforms, limiting our ability to assess temporal or regional differences in content distribution. Fourth, although this study evaluated information quality, completeness, and engagement, it did not directly measure patient comprehension, behavioral change, or clinical outcomes. Fifth, no additional sensitivity analyses were conducted under alternative retrieval orders or sampling strategies, and future studies should further examine the robustness of these findings under different platform-search conditions. Finally, although DISCERN and the related scoring framework were operationally adapted for Chinese short-video assessment through a predefined coding manual, detailed scoring anchors, repeated coder training, and independent evaluation, these tools were originally developed for other health-information formats, and formal validation in Chinese short-video contexts remains limited (49).

Although AI systems and other emerging digital tools may improve access to health information, they cannot replace face-to-face medical assessment, individualized clinical judgment, or physician oversight in matters of patient safety.

Because this was a cross-sectional study, the observed relationships should be interpreted as associations rather than evidence of causal effects or algorithmic mechanisms.

## 5 Conclusion

This cross-sectional study compared the characteristics, information quality, and audience engagement of knee osteoarthritis–related short videos on TikTok, Kwai, and Bilibili in China. The findings demonstrate clear cross-platform differences in digital health information ecosystems. Bilibili provided the most reliable, comprehensive, and educationally valuable content, which may be related to its longer video format and higher proportion of certified medical professionals. In contrast, TikTok generated the highest levels of audience engagement but showed important limitations in information quality and completeness, particularly in relation to etiology and prognosis. Kwai occupied an intermediate position but also showed substantial deficiencies in content rigor.

More importantly, this study identified a consistent inverse relationship between information quality and user engagement. Videos that were more attractive and interaction-oriented tended to achieve greater visibility, whereas videos with stronger scientific rigor and more complete health information were less likely to receive high levels of audience response. This pattern suggests that current platform environments may tend to favor attention-capturing content over evidence-based health communication.

From a public health perspective, these findings highlight an important challenge in the digital communication of chronic disease information. As short-video platforms increasingly serve as major sources of health knowledge for the public, uneven information quality across platforms may affect health literacy, shape disease perceptions, and influence self-management decisions among individuals with knee osteoarthritis or related symptoms. However, information obtained from online platforms should be regarded as supportive rather than sufficient, because it often remains superficial in critical areas such as risk-benefit assessment, individualized recommendations, and recognition of warning signs, and therefore should not be used independently as the primary basis for patient information or clinical decision-making. The results therefore have implications not only for content creators and clinicians, but also for platform governance and the broader regulation of online health information.

To improve the quality and impact of digital public health communication, platform systems should place greater emphasis on the credibility and completeness of health content, while health professionals should adopt more engaging and accessible communication strategies without compromising scientific standards. Future efforts should focus on narrowing the gap between popularity and accuracy so that high-reach health information can also be trustworthy, comprehensive, and supportive of informed chronic disease self-management. While online platforms and emerging AI-based tools may improve access to health information, they cannot replace face-to-face medical assessment, individualized clinical judgment, or established clinical guidelines. The principal contributions of this study to the literature are summarized in Box 1.

Box 1 **Contributions to the literature.**

**Table:** 

This study fills a research gap by systematically comparing knee osteoarthritis information quality across China's three major short-video platforms.
It reveals a critical “popularity-accuracy” trade-off, where high-reach content often lacks scientific rigor and comprehensive coverage.
The findings identify structural deficiencies in etiology and prognosis information, highlighting the risk of fragmented public health knowledge.
The study provides evidence-based strategies for healthcare professionals and platforms to balance engagement with scientific integrity in digital health communication.

## Data Availability

The raw data supporting the conclusions of this article will be made available by the authors, without undue reservation.

## References

[1] Steinmetz JD Culbreth GT Haile LM Rafferty Q Lo J Fukutaki KG. Global, regional, and national burden of osteoarthritis, 1990–2020 and projections to 2050: a systematic analysis for the global burden of disease study 2021. Lancet Rheumatol. (2023) 5(9):e508–22. 10.1016/S2665-9913(23)00163-7 37675071 PMC10477960

[2] Cui A Li H Wang D Zhong J Chen Y Lu H. Global, regional prevalence, incidence and risk factors of knee osteoarthritis in population-based studies. eClinicalMed. (2020) 29-30:100587. 10.1016/j.eclinm.2020.100587 PMC7704420 34505846

[3] Vina ER Kwoh CK. Kwoh CK: epidemiology of osteoarthritis: literature update. Curr Opin Rheumatol. (2018) 30(2):160–7. 10.1097/BOR.0000000000000479 29227353 PMC5832048

[4] Almalki A Gharib AF Almehmadi M Alharthi A Alsalmi O Alsulimani AH. The association of vitamin D, growth/differentiation factor 5 (GDF-5) gene polymorphism, and Serum GDF-5 protein in obese patients with knee osteoarthritis. Cureus. (2023) 15(11):e48350. 10.7759/cureus.48350 38060707 PMC10698833

[5] Wang YP Di WJ Yang S Qin SL Xu YF Han PF. The association of growth differentiation factor 5 rs143383 gene polymorphism with osteoarthritis: a systematic review and meta-analysis. J Orthop Surg Res. (2023) 18(1):763. 10.1186/s13018-023-04245-y 37817264 PMC10563324

[6] Hunter DJ Bierma-Zeinstra S. Osteoarthritis. Lancet. (2019) 393(10182):1745–59. 10.1016/S0140-6736(19)30417-9 31034380

[7] Fugaru FO Kamal A Kamal D Traşcă D Kamal C Trăistaru M. Rehabilitation in knee osteoarthritis patients in 2020. Narrative review. Health Sports Rehabil Med. (2021) 12(2):34–9. 10.26659/pm3.2021.22.2.100

[8] Zeng Z Shan J Zhang Y Wang Y Li C Li J. Asymmetries and relationships between muscle strength, proprioception, biomechanics, and postural stability in patients with unilateral knee osteoarthritis. Front Bioeng Biotechnol. (2022) 10:922832. 10.3389/fbioe.2022.922832 36185430 PMC9523444

[9] Katz JN Arant KR Loeser RF. Diagnosis and treatment of hip and knee osteoarthritis: a review. JAMA. (2021) 325(6):568–78. 10.1001/jama.2020.22171 33560326 PMC8225295

[10] Aratikatla A Maffulli N Gupta M Potti IA Potty AG Gupta A. Wharton’s jelly and osteoarthritis of the knee. Br Med Bull. (2024) 149(1):13–31. 10.1093/bmb/ldad030 38061765 PMC10993459

[11] Budarick AR Moyer RF. Linking physical activity with clinical, functional, and structural outcomes: an evidence map using the osteoarthritis initiative. Clin Rheumatol. (2022) 41(4):965–75. 10.1007/s10067-021-05995-y 34802082

[12] Gibbs AJ Gray B Wallis JA Taylor NF Kemp JL Hunter DJ. Recommendations for the management of hip and knee osteoarthritis: a systematic review of clinical practice guidelines. Osteoarthritis Cartilage. (2023) 31(10):1280–92. 10.1016/j.joca.2023.05.015 37394226

[13] Zhang C Zhu B. Digital gratification: short video consumption and mental health in rural China. Front Public Health. (2025) 13:1536191. 10.3389/fpubh.2025.1536191 40331122 PMC12052899

[14] Zuo QH Du K Li A Zhang CY Guo R Chen P. Quality and reliability of osteoarthritis-related health information on short video platforms: a cross-sectional comparative study of TikTok and Bilibili. Front Digit Health. (2025) 7:1623247. 10.3389/fdgth.2025.1623247 41281294 PMC12634650

[15] Tu J Zhang C Zhang H Liang L He J. Evaluating the reliability and quality of knee osteoarthritis educational content on TikTok and Bilibili: a cross-sectional content analysis. Digit Health. (2025) 11:20552076251366390. 10.1177/20552076251366390 40808711 PMC12344336

[16] Selberg AM Pei M Nguyen HN Bosland A Beebe KS. Tiktok, #kneepain, and medical (mis)information. J Orthop Rep. (2025) 4(3):100405. 10.1016/j.jorep.2024.100405

[17] Priyambodo AZ Zhafirah NA Shabur R Handayani PW Fitriani H. Health information-seeking using short video platforms. Digit Health. (2025) 11:20552076251394619. 10.1177/20552076251394619

[18] Crespo-Gonzalez C Benrimoj SI Frommer M Dineen-Griffin S. Navigating online health information: insights into consumer influence and decision-making strategies-an overview of reviews. Digit Health. (2024) 10:20552076241286815. 10.1177/20552076241286815 39493637 PMC11528751

[19] Kjærulff EM Andersen TH Kingod N Nexø MA. When people with chronic conditions turn to peers on social Media to obtain and share information: systematic review of the implications for relationships with health care professionals. J Med Internet Res. (2023) 25:e41156. 10.2196/41156 PMC10152331 37067874

[20] Feyzioğlu Ö Öztürk Ö. Quality and compliance of video-based information with knee osteoarthritis treatment guidelines. J Basic Clin Health Sci. (2024) 8(2):390–401. 10.30621/jbachs.1340729

[21] Zhang R Zhang Z Jie H Guo Y Liu Y Yang Y. Analyzing dissemination, quality, and reliability of Chinese brain tumor-related short videos on TikTok and Bilibili: a cross-sectional study. Front Neurol. (2024) 15:1404038. 10.3389/fneur.2024.1404038 39494168 PMC11527622

[22] Zeng F Zhang W Wang M Zhang H Zhu X Hu H. Tiktok and Bilibili as sources of information on lung cancer in China through assessment and analysis of the content and quality. Sci Rep. (2024) 14(1):20604. 10.1038/s41598-024-70640-y 39232044 PMC11375008

[23] Zhu W He B Wang X Du Y Young K Jiang S. Information quality of videos related to esophageal cancer on TikTok, Kwai, and Bilibili: a cross-sectional study. BMC Public Health. (2025) 25(1):2245. 10.1186/s12889-025-23475-9 40604838 PMC12220660

[24] Guan JL Xia SH Zhao K Feng LN Han YY Li JY. Videos in short-video sharing platforms as sources of information on colorectal polyps: cross-sectional content analysis study. J Med Internet Res. (2024) 26:e51655. 10.2196/51655 39470708 PMC11558218

[25] Wang L Chen Y Zhao D Xu T Hua F. Quality and dissemination of uterine fibr oid health information on TikTok and Bilibili: cross-sectional study. JMIR Form Res. (2025):9:e75120. 10.2196/75120 40749219 PMC12316441

[26] DemandSage. How many people use TikTok in 2024? Statistics and facts. (2024). Available online at: https://www.demandsage.com/tiktok-user-statistics/ (accessed December 9, 2024).

[27] iiMedia Research. Survey data on user behavior in China’s short video industry. (2023). Available online at: https://www.iimedia.cn/c1077/97043.html (accessed December 9, 2024).

[28] Bernard A Langille M Hughes S Rose C Leddin D Veldhuyzen van Zanten S. A systematic review of patient inflammatory bowel disease information resources on the world wide web. Am J Gastroenterol. (2007) 102(9):2070–7. 10.1111/j.1572-0241.2007.01325.x 17511753

[29] Charnock D Shepperd S Needham G Gann R. DISCERN: an instrument for judging the quality of written consumer health information on treatment choices. J Epidemiol Commun Health. (1999) 53(2):105–11. 10.1136/jech.53.2.105 PMC1756830 10396471

[30] Zheng S Tong X Wan D Hu C Hu Q Ke Q. Quality and reliability of liver CancerRelated short Chinese videos on TikTok and Bilibili: cross-sectional content analysis study. J Med Internet Res. (2023) 25:e47210. 10.2196/47210 37405825 PMC10357314

[31] DISCERN. The DISCERN Handbook: Quality Criteria for Consumer Health Information on Treatment Choices. Abingdon: Radcliffe Medical Press (1998).

[32] Hu Y Yang Y Chen N Pang Z Zhong X Sun J. How to select high-quality health educational short videos on social media? Insights from YouTube and TikTok. BMC Med Educ. (2025) 25(1):1418. 10.1186/s12909-025-08018-5 41088163 PMC12523207

[33] Sun M Zang D Chen J. Cross-platform assessment of short-form video quality on the gut-liver axis: informational integrity and engagement disparity. Front Cell Infect Microbiol. (2025) 15:1732375. 10.3389/fcimb.2025.1732375 41450569 PMC12727577

[34] Zhang X Kamarudin S Tang QQ Liu XT. Scroll, watch, engage? A data-driven content analysis of celebrity physicians’ health video strategies on TikTok. J Inf Commun Ethics Soc. (2025) 23(3):392–414. 10.1108/JICES-02-2025-0037

[35] Xiao L Sun L Lei Z Fu B. How do features in health short-form videos impact viewer engagement: an empirical study. Ind Manag Data Syst. (2026) 126(2):678–709. 10.1108/IMDS-04-2024-0385

[36] Li B Liu M Liu J Zhang Y Yang W Xie L. Quality assessment of health science-related short videos on TikTok: a scoping review. Int J Med Inform. (2024) 186:105426. 10.1016/j.ijmedinf.2024.105426 38531256

[37] Zhang C Liu S Zhou X Zhou S Tian Y Wang S. Examining the role of large language models in orthopedics: systematic review. J Med Internet Res. (2024) 26:e59607. 10.2196/59607 39546795 PMC11607553

[38] Duan X Wang Y Ma T Cai M Ma J Ma X. Quality and reliability of knee osteoarthritis-related information on short video platforms in China: a multi-method cross-sectional study. BMC Public Health. (2026) 26:770. 10.1186/s12889-026-26455-9 41629905 PMC12955160

[39] Yurttutmuş Tatlı ZR Ferahman D. YouTube As a source of information on thoracic outlet syndrome: quality and reliability analysis. Eur Res J. (2025) 11(3):564–73. 10.18621/eurj.1596399

[40] Fahy S Oehme S Milinkovic DD Bartek B. Enhancing patient education on the role of tibial osteotomy in the management of knee osteoarthritis using a customized ChatGPT: a readability and quality assessment. Front Digit Health. (2025) 6:1480381. 10.3389/fdgth.2024.1480381 39830641 PMC11738919

[41] O'Donnell N Jerin SI Mu D. Using TikTok to educate, influence, or inspire? A content analysis of health-related EduTok videos. J Health Commun. (2023) 28(8):539–51. 10.1080/10810730.2023.2234866 37434532

[42] Wang L Li Y He J. Dissemination of obesity-related short videos on TikTok: content analysis. Discov Public Health. (2025) 22:166. 10.1186/s12982-025-00560-3

[43] Zhang K Li Z Lin Y Guo Y Huang R Liu G. Bilibili, TikTok and Xiaohongshu as health information platforms for stroke: evaluating information quality and content. Sci Rep. (2025) 15(1):37705. 10.1038/s41598-025-21535-z 41152378 PMC12568935

[44] Guo F Ding G Zhang Y Liu X. Quality assessment of radiotherapy health information on short-form video platforms of TikTok and Bilibili: cross-sectional study. JMIR Cancer. (2025) 11:e73455. 10.2196/73455 40986789 PMC12456845

[45] Kington RS Arnesen S Chou WYS Curry SJ Lazer D Villarruel AM. Identifying credible sources of health information in social media: principles and attributes. NAM Perspect. (2021) 2021. 10.31478/202107a 34611600 PMC8486420

[46] Ghalavand H Nabiolahi A. Exploring online health information quality criteria on social media: a mixed method approach. BMC Health Serv Res. (2024) 24(1):1311. 10.1186/s12913-024-11838-8 39478573 PMC11523579

[47] Mohamed H Kittle E Nour N Hamed R Feeney K Salsberg J. An integrative systematic review on interventions to improve layperson’s ability to identify trustworthy digital health information. PLOS Digit Health. (2024) 3(10):e0000638. 10.1371/journal.pdig.0000638 39453891 PMC11508166

[48] Akdere E Karpuz S Yılmaz R Yılmaz H Solak İ. Evaluation of readability and reliability of turkish websites on low back pain. Ege Tıp Bilimleri Dergisi. (2024) 7(1):18–23. 10.33713/egetbd.1444781.

[49] Du K Li A Zuo QH Zhang CY Guo R Chen P. Comparing artificial intelligence-generated and clinician-created personalized self-management guidance for patients with knee osteoarthritis: blinded observational study. J Med Internet Res. (2025) 27:e67830. 10.2196/67830 40332991 PMC12096024

